# Avian Assemblages at Bird Baths: A Comparison of Urban and Rural Bird Baths in Australia

**DOI:** 10.1371/journal.pone.0150899

**Published:** 2016-03-10

**Authors:** Gráinne P. Cleary, Holly Parsons, Adrian Davis, Bill R. Coleman, Darryl N. Jones, Kelly K. Miller, Michael A. Weston

**Affiliations:** 1 National Parks Association of New South Wales, PO Box 337, Newtown, New South Wales, Australia; 2 Deakin University, Geelong, Australia. Centre for Integrative Ecology, School of Life and Environmental Sciences, Burwood Campus, Burwood, Victoria, Australia; 3 BirdLife Australia, Sydney Olympic Park, New South Wales, Australia; 4 The University of Sydney, School of Biological Sciences, Botany Annex A13, Sydney, New South Wales, Australia; 5 Evolve Information Services, Level 8, Melbourne, Victoria, Australia; 6 Environmental Futures Research Institute, Griffith University, Nathan, Queensland, Australia; University of Minnesota, UNITED STATES

## Abstract

Private gardens provide habitat and resources for many birds living in human-dominated landscapes. While wild bird feeding is recognised as one of the most popular forms of human-wildlife interaction, almost nothing is known about the use of bird baths. This citizen science initiative explores avian assemblages at bird baths in private gardens in south-eastern Australia and how this differs with respect to levels of urbanisation and bioregion. Overall, 992 citizen scientists collected data over two, four-week survey periods during winter 2014 and summer 2015 (43% participated in both years). Avian assemblages at urban and rural bird baths differed between bioregions with aggressive nectar-eating species influenced the avian assemblages visiting urban bird baths in South Eastern Queensland, NSW North Coast and Sydney Basin while introduced birds contributed to differences in South Western Slopes, Southern Volcanic Plains and Victorian Midlands. Small honeyeaters and other small native birds occurred less often at urban bird baths compared to rural bird baths. Our results suggest that differences between urban versus rural areas, as well as bioregion, significantly influence the composition of avian assemblages visiting bird baths in private gardens. We also demonstrate that citizen science monitoring of fixed survey sites such as bird baths is a useful tool in understanding large-scale patterns in avian assemblages which requires a vast amount of data to be collected across broad areas.

## Introduction

In almost every continent, urbanisation has transformed landscapes for humans and wildlife with much of this transformation occurring over the last few centuries. Australian cities, for example, have been transformed from pristine habitat to highly modified urban environments in only c. 220 years [1]. As the world becomes increasingly urbanised, understanding how to conserve biodiversity in increasingly modified and novel environments has become a major conservation challenge [2]. Additionally, as more people inhabit cities, the physical and psychological disconnect between people and the natural environment is likely to increase [3]. When planned and managed correctly urban areas offer the potential to maintain or create habitat for wildlife and also connect people with nature [4]. To address these challenges, urban ecology has received increased attention from scientists interested in understanding the influence of human activities on urban ecosystems and their ecological communities [5] by comparing biodiversity between urban and rural habitats [6].

Urbanisation is one of the leading causes of species extinction due to extensive habitat alteration [3]. Anthropogenic modifications alter avian assemblages, typically resulting in a reduction of biodiversity as many birds are unable to persist and are forced to either adapt, move or face local extirpation [7, 8, 9]. As a result, urbanisation can promote simplification of avian assemblages [10] with an increase in abundance of a few species able to persist within, or adapt to, the urban environment [7]. Such birds are usually larger-bodied, dominant birds, often with generalist or omnivorous diets, and are referred to as “urban adapters” and/or “exploiters” [11]. Many of these species are also introduced species, which can replace native species, thus promoting biotic homogenisation at various spatial scales [11, 12, 13]. Birds introduced into Australia include House Sparrows (*Passer domesticus*), Common Mynas (*Acridotheres tristis*), Common Starlings (*Sturnis vulgaris*), Common Blackbirds (*Turdus merula*) and Spotted Doves (*Streptopelia chinensis*). These introduced birds appear to be persisting in urban habitats, utilising anthropogenic features such as buildings, paved areas and lawns for feeding, roosting and nesting resources [14, 15]. In addition to introduced species, certain native nectarivorous species have responded positively to urbanisation and have become a dominant component of the urban avifauna [16]. Noisy Miners (*Manorina melanocephala*) and Red Wattlebirds (*Anthochaera carunculata*), two large honeyeaters, have increased throughout several major Australian cities since European settlement [17, 18, 19] and proved to be a successful group of avian urban adaptors [20, 21]. Urbanised environments have similar anthropogenic features regardless of the prevailing biogeography and can favour species with certain biological and life-history traits advantageous for living in fragmented and novel environments [22]. Although similar traits are evident among urban adapters and exploiters, cities occur within different biogeographical contexts, so species pools may differ between cities; this has not, however, been studied at larger scales such as continental scales.

One valuable approach to investigating the effects of urbanisation involves comparisons of avifauna in rural and urban areas. Such studies have typically involved comparisons of sites within single biogeographical regions and most have demonstrated that urbanisation homogenises bird assemblages [11, 13, 23, 24]. However, comparisons derived from larger geographical scales (i.e. across bioregions) and which control for the influence of habitat-mediated detection probabilities remain scarce [25, 26]. Such studies are necessary if we are to understand how urbanisation influences birds more generally [27].

Many households provide attractants such as food, water for bathing or drinking and/or shelter resources for birds in the form of bird feeders, nest boxes and water sources (‘bird baths’) and thus may influence the abundance and diversity of bird species in their gardens [22, 28]. Surveying attractants such as bird baths represents a useful opportunity for understanding bird assemblages in their vicinity especially in the dry continent of Australia. Australia is particularly susceptible to multi-year dry episodes [29] and it is expected that it will experience generally drier conditions in the future [30]. Drought can result in long-term changes to habitat and resources with Australia regularly experiencing drought cycles lasting ten years or more [31, 32]. Therefore, the provision of supplementary water may support otherwise stressed bird populations. Indeed, it has been reported that decreases in inland rainfall resulted in an increase in abundance of parrots in the urban landscape that traditionally inhabited inland areas [33]. Despite this, the provision of water to birds has received virtually no research attention (but see [34]). Bird baths are interesting in their own right because of the growing prominence of the provision of wildlife attractants and controversy with respect to whether they represent conservation benefits or problems [35, 36]. Conservation benefits to birds might include a supply of clean fresh water while problems include attracting introduced species, creating overdependence upon an unreliable or inadequate water supply, and the potential risk of disease [28, 37].

We conducted a citizen science study monitoring birds visiting bird baths over two seasons (winter 2014 and summer 2015) to investigated bird assemblages using bird baths in urban and rural areas, across bioregions of Australia. We tested if bird assemblages at urban and rural bird baths differ *across* bioregions and if bird assemblages at urban and rural bird baths differ *within* bioregions. We were interested in testing for differences in species richness at urban bird baths compared to rural bird baths. As far as we are aware, this is the first study to compare urban and rural avian assemblages in private gardens at a large spatial scale, across different biogeographical regions, using data gathered by citizen scientists monitoring bird baths.

## Methods

Data on bird occurrence at bird baths were collected during “The Bathing Birds Study” that ran for a four week period in each of two seasons: austral winter (June 24^th^ to July 26^th^ 2014) and summer (January 27^th^ to February 29^th^ 2015). The study was promoted throughout Australia to recruit citizen scientists via: (1) media coverage (television, radio and newspapers), (2) social media (particularly via Facebook), (3) communication networks of a range of project partners, and (4) by targeting specific ornithological associations across Australia. Participants used an on-line data portal hosted on the *Atlas of Living Australia* (ALA) website (ala.org.au) to report the presence of birds visiting their bird bath during the survey. Other data collected included location of the bird bath, number of bird visits and photographs for validation of sightings. To aid participants with identification, an online field guide was available and participants could email photo and descriptions of birds to aid identification.

During the two survey periods, citizen scientists monitored their bird baths for 20 minutes, once per day and three times per week for four weeks (surveys which did not meet these criteria were not considered further) to detect all or most species visiting bird baths [38]. Due to the difficulty in accurately surveying birds in rain or high winds, we asked our citizen scientists to conduct surveys in relatively calm and rain-free weather. Data were pooled (sightings of each species across all surveys with each survey period) within each bird bath and expressed as a binary indicator of whether a given species was present or absent at a bird bath. Due to limitations of the technology platform used to collect data, we were only able to record occurrence/presence of birds at baths. It was not possible to capture surveys where there were no sightings present although such surveys are regarded as highly unlikely. Bird baths were assigned to:

1. Bioregion (Interim Biogeographic Regionalisation for Australia) regions, henceforth ‘bioregion’, a classification based on climate, vegetation and soil (National Land and Water Resources Audit, 2001). Bird data were collected from 42 bioregions but due to low participation (< 3 participants in an urban or rural area), assessment of differences were conducted on 8 (winter) and 13 (summer) bioregions.
2. “Rural” or “urban” areas according to the Australian Bureau of Statistics (ABS) classification which uses an Australian Statistical Geography Standard that defines “urban” areas as having human populations of 1,000–100,000+ people while “rural” areas have < 999 people. Thus, rural areas may contain large natural areas as well as low-density human settlement.

### Data Analysis

Winter and summer survey periods were analysed separately and each bird bath was treated as an independent replicate for all analyses. We determined the presence/absence of each bird species at urban and rural bird baths.

#### Question 1: Do assemblages at urban and rural bird baths differ across bioregions?

To test for differences between bird assemblages at rural and urban bird baths we first examined whether bioregion affected assemblages and any differences between urban and rural bird baths using PERMANOVA. Bioregion was included as a random factor and urbanisation (urban or rural) type as a fixed two-level factor (urban versus rural).

#### Question 2: Do assemblages at urban and rural bird baths differ within bioregions?

Assemblage composition at urban and rural bird baths was visualised for each bioregion (for which sufficient data were available) using non-metric Multidimensional Scaling (nMDS) ordination techniques. PERMANOVA pair-wise comparisons tested for assemblage composition differences between urban and rural bird baths for each bioregion. Similarity percentage analysis (SIMPER) was used to identify species contributions to any dissimilarities between rural and urban bird baths for bioregions where pair-wise comparisons suggested significant differences existed.

#### Question 3: Is species richness different at bird baths in rural areas compared to urban areas?

Differences in species richness between urban and rural bird baths by bioregion were compared using Mann-Whitney U tests.

## Results

**Fig 1:**
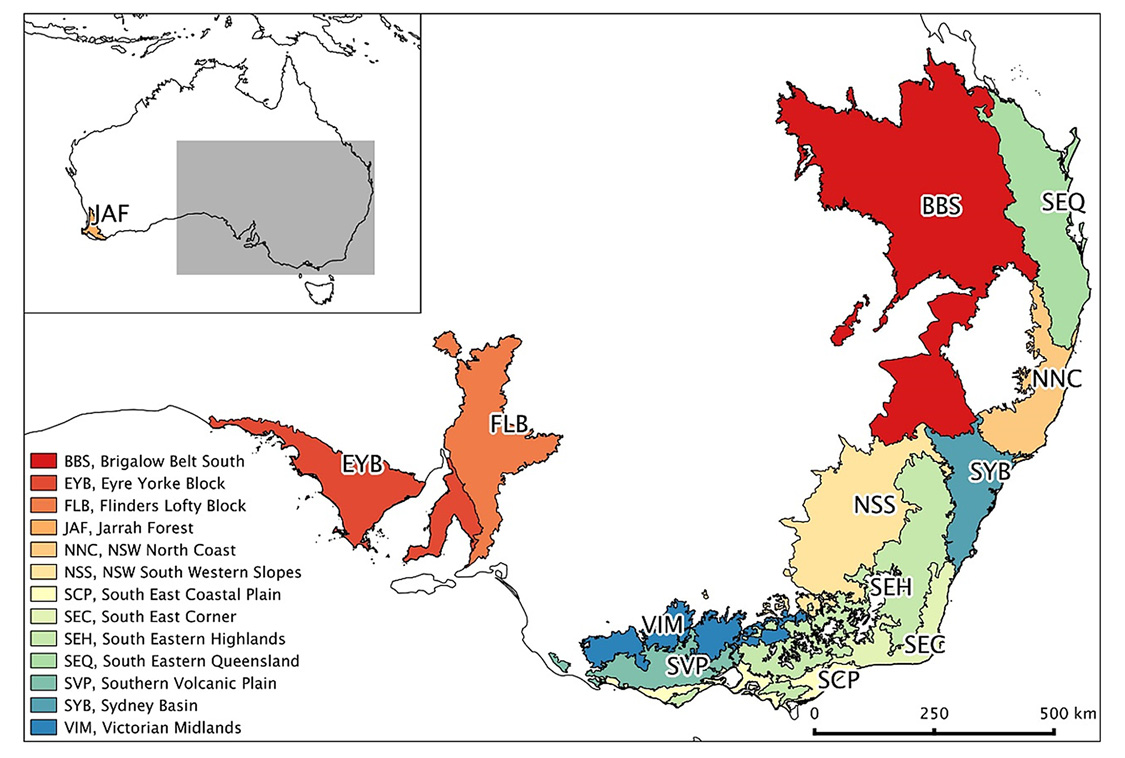
Map depicting the bioregions referred to in Table 2.

Significant differences in bird assemblages were detected at bird baths between bioregions and between urban and rural areas (Table 1). In the summer survey ten bioregions (Brigalow Belt South, Flinders Lofty Block, NSW North Coast, NSW South Western Slopes, South East Coastal Plain, South Eastern Highlands, South Eastern Queensland, Southern Volcanic Plain, Sydney Basin, Victorian Midlands) were identified as having differences in bird communities at bird baths between urban and rural areas. For the winter survey three of these bioregions (South East Queensland, NSW North Coast and Sydney Basin) exhibited differences, thus for these bioregions differences were evident in both seasons (Fig 1, Table 2).

**Table 1:** Two PERMANOVAs (one per survey period) of bird assemblages at bird baths between bioregions and urban versus rural areas.

Season	Source	Type	DF	Pseudo F	P (perms.)	Unique Perms.
**Winter**	Bioregion	Random	8	4.8879	0.001*	999
	Urbanisation	Fixed	1	5.235	0.006*	998
**Summer**	Bioregion	Random	12	5.2373	0.001*	995
	Urbanisation	Fixed	1	9.6009	0.001*	998

Statistically significant results are indicated by *.

**Table 2:** PERMANOVA pair-wise comparisons of bird assemblages at bird baths in urban areas and rural areas for individual bioregions. Different PERMANOVA pair-wise comparisons between urban and rural assemblages were conducted for winter and summer. ‘-‘ indicates < 3 participants in urban and/or rural categories within a bioregion, thus precluding analysis.

Bioregion	Winter T Statistic	Summer T Statistic
Brigalow Belt South (BBS)	1.1744	1.3648*
Eyre Yorke Block (EYB)	-	0.9691
Flinders Lofty Block (FLB)	-	1.6599*
Jarrah Forest (JAF)	-	1.0895
NSW North Coast (NNC)	1.7962*	1.5389*
NSW South Western Slopes (NSS)	1.2548	1.4285*
South East Coastal Plain (SCP)	1.2494	1.9405*
South East Corner (SEC)	1.1037	1.0332
South Eastern Highlands (SEH)	1.2870	2.1076*
South Eastern Queensland (SEQ)	1.9755*	1.7544*
Southern Volcanic Plains (SVP)	-	1.9985*
Sydney Basin (SYB)	4.0132*	3.2107*
Victorian Midlands (VIM)	1.2402	1.8211*

Statistically significant results are indicated by *.

**Fig 2:**
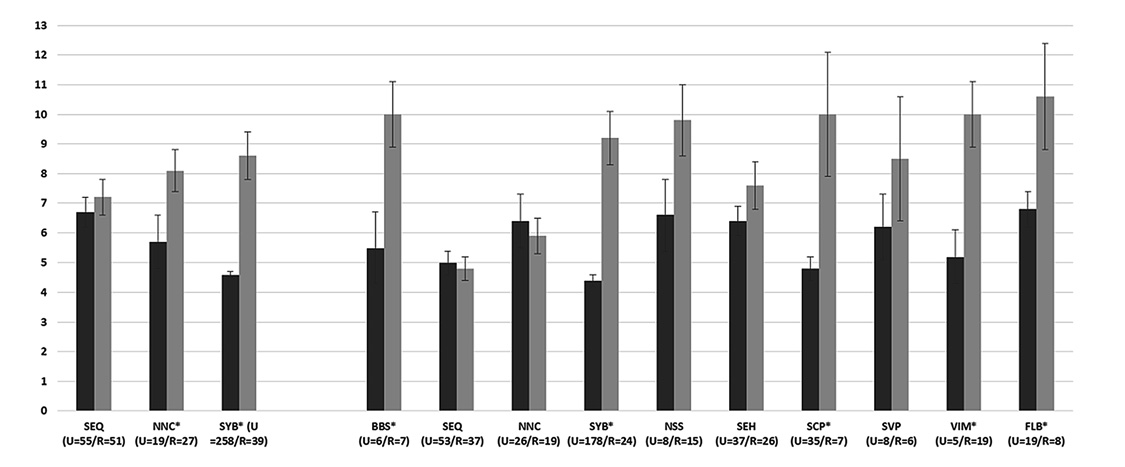
The number of bird baths monitored in urban (U, black bars) and rural (R, grey bars) (U = n/R = n) areas and mean bird species richness (± SE) for each bioregion in the winter survey (SYB, NNC, SEQ) and summer survey (BBS, SEQ, NNC, SYB, NSS, SEH, SCP, SVP, VIM, FLB). The significance of a comparison of species richness per bird bath between urban and rural baths is presented (Mann-Whitney U test, statistically significant results are indicated by *). Bioregions are abbreviated, see Table 2 and Fig 1 for full names).

A total of 449 bird baths were monitored during the winter survey period from 3 bioregions and 543 bird baths in summer from 10 bioregions; 43% of citizen scientists participated in both winter and summer surveys. Overall, 73% and 69% of citizen scientists took part from urban areas and 26.9% and 30.9% from rural areas, in winter and summer, respectively (Fig 2).

For the winter survey period, 147 bird species (19 223 records) were recorded at bird baths. Native species were the most commonly observed species at bird baths in both seasons with Rainbow Lorikeets (*Trichoglossus haematodus*) reported at 29% of bird baths, followed by Noisy Miners 28%) and Australian Magpies (*Cracticus tibicen*; 24%). In summer, 172 bird species (22 377 records) were reported at bird baths. Noisy Miners were the most frequently reported bird occurring at 32% of bird baths followed by Australian Magpies (31%) and Red Wattlebirds (*Anthochaera carunculata*; 22%).

For the winter survey, species richness at bird baths was significantly higher at rural compared to urban bird baths in two bioregions: New South Wales North Coast and Sydney Basin (Fig 2). In summer, species richness was significantly higher at rural bird baths in five bioregions: Brigalow Belt South, Sydney Basin, South East Coastal Plains, Victorian Midlands and Flinders Lofty Block (Fig 2). Different species contributed to bird assemblages between urban and rural bird baths depending on bioregion and season. Across the whole data set, higher richness occurred at rural bird baths in both seasons (winter, Mann-Whitney U = 10946.0, Z = -6.438. P < 0.001; summer, Mann-Whitney U = 22155.0, Z = -6.438, P < 0.001).

Nectar-eating birds contributed to the differences in assemblages at bird baths in both the winter and summer surveys. Depending on bioregion, different nectar-eating birds contributed to differences. Three aggressive honeyeaters contributed to differences in bird assemblages at urban bird baths compared to rural; Noisy Miners and Rainbow Lorikeets contributed substantially to dissimilarity in three bioregions (South-eastern Queensland, NSW North Coast and Sydney Basin) (Tables 3 and 4).). Red Wattlebirds contributed to differences in bird assemblages at both rural and urban bird baths in two bioregions, South Eastern Highlands and South East Coastal Plains during the summer survey (Table 4).

**Table 3:** SIMPER (similarity percentages) analysis of bird species that contributed ≥ 4.5% to the Bray-Curtis indices of dissimilarity between avian assemblages at urban and rural bird baths during the winter survey. Proportion occurrence represents the proportion of bird baths at which a given species was recorded.

Bioregion (Average dissimilarity)	Species	Proportion Occurrence Urban	Proportion Occurrence Rural	Contrib. %	Cumul. %
**South East Queensland (85.56)**	Noisy Miner *Manorina melanocephala*	0.47	0.39	5.11	5.11
	Lewin’s Honeyeater*Meliphaga lewinii*	0.24	0.53	4.84	9.96
	Rainbow Lorikeet *Trichoglossus haematodus*	0.42	0.31	4.69	14.65
**NSW North Coast (87.27)**	Lewin’s Honeyeater*Meliphaga lewinii*	0.21	0.59	5.06	5.06
	Rainbow Lorikeet*Trichoglossus haematodus*	0.53	0.22	4.86	9.92
**Sydney Basin (91.24)**	Eastern Spinebill*Acanthorhynchus tenuirostris*	0.10	0.50	4.14*	4.14

*Highest contribution percentage.

**Table 4:** SIMPER analysis of bird species that contributed ≥ 4.5% or more to the Bray-Curtis indices of dissimilarity between bird assemblages at urban and rural bird baths during the summer survey. Proportion occurrence represents the proportion of bird baths at which a given species was recorded.

Bioregion (Average dissimilarity)	Species	Proportion Occurrence Urban	Proportion Occurrence Rural	Contrib. %	Cumul. %
**Brigalow Belt South (87.14)**	Double-barred Finch *Taeniopygia bichenovii*	0.17	0.86	5.87	5.87
**South Eastern Queensland (88.89)**	Noisy Miner *Manorina melanocephala*	0.51	0.32	7.19	7.19
	Lewin’s Honeyeater *Meliphaga lewinii*	0.13	0.49	6.35	13.54
**NSW North Coast (88.83)**	Satin Bowerbird *Ptilonorhynchus violaceus*	0.35	0.37	5.22	5.22
	Noisy Miner *Manorina melanocephala*	0.46	0.16	4.66	9.88
**Sydney Basin (90.58)**	Red-browed Finch *Neochmia temporalis*	0.06	0.57	4.84	4.84
	Noisy Miner *Manorina melanocephala*	0.48	0.22	4.84	9.68
**South Western Slopes (82.57)**	^Common Blackbird*Turdus merula*	0.63	0.13	5.12	5.12
	Superb Fairy-wren *Malurus cyaneus*	0.25	0.69	4.88	10.01
	Australian Magpie *Cracticus tibicen*	0.25	0.56	4.63	14.64
**South Eastern Highlands (84.94)**	Grey Fantail *Rhipidura albiscapa*	0.14	0.62	4.95	4.95
	Red Wattlebird *Anthochaera carunculata*	0.57	0.19	4.94	9.88
	Crimson Rosella *Platycercus elegans*	0.41	0.5	4.74	14.63
	Superb Fairy-wren *Malurus cyaneus*	0.22	0.54	4.63	19.26
**South East Coastal Plain (86.25)**	Superb Fairy-wren *Malurus cyaneus*	0.06	0.71	5.47	5.47
	Grey Fantail *Rhipidura albiscapa*	0.00	0.57	5.29	10.76
	Red Wattlebird *Anthochaera carunculata*	0.40	0.57	4.61	15.76
	Australian Magpie *Cracticus tibicen*	0.46	0.29	4.51	19.87
**Southern Volcanic Plains (85.58)**	^Spotted Dove *Streptopelia chinensis*	0.88	0.00	8.40	8.40
	Superb Fairy-wren *Malurus cyaneus*	0.25	0.83	5.68	14.07
	^Common Blackbird *Turdus merula*	0.50	0.33	5.43	19.50
	New Holland Honeyeater *Phylidonyris novaehollandiae*	0.50	0.67	4.67	24.18
	^House Sparrow *Passer domesticus*	0.63	0.50	4.67	28.84
**Victorian Midlands (84.75)**	Superb Fairy-wren *Malurus cyaneus*	0.00	0.79	6.20	6.20
	Crimson Rosella *Platycercus elegans*	0.00	0.63	5.45	11.66
	^Common Myna *Acridotheres tristis*	0.60	0.00	4.89	16.55
**Flinders Lofty Block (76.55)**	Eastern Spinebill *Acanthorhynchus tenuirostris*	0.11	0.63	5.03	5.03

^ indicates introduced birds.

While these large and dominant nectar-eating birds drove differences at urban bird baths, three smaller nectar-eating birds contributed to differences at rural bird baths. Lewin’s Honeyeaters contributed to dissimilarities in bird assemblages at bird baths with a higher occurrences at rural bird baths in two bioregions, South Eastern Queensland in both seasons and in NSW North Coast during the winter study (Tables 3 and 4). New Holland Honeyeaters and Eastern Spinebills contributed to differences between assemblages, appearing in high occurrence at rural baths in three bioregions in the winter and in the summer survey in Southern Volcanic Plains and Flinders Lofty Block (Tables 3 and 4).

During the summer survey, four species of small native birds, Superb Fairy-wrens, Grey Fantails, Red-browed Finches and Double-barred Finches contributed to differences between assemblages, with a high occurrence at rural bird baths in seven bioregions (Tables 3 and 4). Superb Fairy-wrens were recorded frequently at rural bird baths in five bioregions. In two bioregions both Superb Fairy-wrens and Grey Fantails were identified as contributing to differences in assemblages with a higher abundance at rural bird baths compared to urban baths (Tables 3 and 4).

Introduced birds contributed to dissimilarities in bird assemblages in three bioregions where they were recorded in a high occurrence at urban bird baths: South Western Slopes, Southern Volcanic Plains and Victorian Midlands during the summer survey (Table 3). In Southern Volcanic Plains three species of introduced birds contributed to Bray-Curtis average dissimilarities index at urban bird baths: Spotted Doves, Common Blackbirds and House Sparrows (Table 3).

## Discussion

Avian assemblages differed between bioregions and between urban and rural areas with particular species contributing differences (55% of species contributed to differences over > 1 bioregion). Birds that drove differences at bird baths in three the more northerly bioregions (South Eastern Queensland, NSW North Coast and Sydney Basin) were similar to each other (high occurrence of nectar-eating birds) but were distinct from more southerly bioregions (South Western Slopes, South Eastern Highlands, South East Coastal Plain Southern Volcanic Plains and Victorian Midlands) where species which drove differences were predominantly a mix of introduced birds, native generalist and nectarivorous birds. Species richness at bird baths was higher at rural bird baths compared to urban bird baths across the whole data set and in a number of bioregions; in winter species richness was higher at rural bird baths in NSW North Coast and Sydney Basin and in summer at rural bird baths in Brigalow Belt South, Sydney Basin, South East Coastal Plains, Victorian Midlands and Flinders Lofty Block.

In South Eastern Queensland, NSW North Coast and Sydney Basin, Noisy Miners and Rainbow Lorikeets were dominant at urban bird baths while in more southern bioregions (South East Coastal Plain, South Western Slopes, Southern Volcanic Plains and Victorian Midlands) introduced birds (Common Blackbirds, Spotted Doves and House Sparrows) were more prevalent at urban bird baths. Our data aligns with other work [21] which has suggested that in more northerly bioregions, aggressive nectar-eating birds appear to be making use of urban resources or have remained in the landscape as it becomes more urbanised. The same can be said for introduced birds with a high occurrence at urban bird baths compared to rural bird baths. This may be attributed to many of these species being adapted to human-dominated landscapes [15, 39]. For example, three introduced birds were identified as drivers at urban birdbaths in the Southern Volcanic Plains: Spotted Doves, House Sparrows and Common Blackbirds. Our results show that introduced birds substantially altered assemblage composition between urban and rural areas in some more southerly bioregions, while they did not in more northerly bioregions (South Eastern Queensland, NSW North Coast and Sydney Basin). While invasive introduced species are usually highly adaptable and ecologically flexible, they have climatic and habitat tolerances [40,41].

Noisy Miners and Rainbow Lorikeets appear to be doing well in urban areas by exploiting nectar rich resources, particularly large hybrid Grevillea species that are popular in urban gardens, parks and streets [42,43]. Rainbow Lorikeets were previously absent in cities in the early 1900’s, but are now observed in densities between 1.67 birds per hectare (Melbourne) and 8 birds per hectare (Townsville) [44,45,46,47] and are now one of the most frequently recorded species in Sydney [20]. In addition urban populations of nectar-eating birds may appear to be periodically boosted by environmental stresses such as bushfire or drought and may exhibit a seasonal shift in abundance [33, 45]. For example, Rainbow Lorikeets were identified as contributing to assemblages at bird baths in South East Queensland and NSW North Coast during the winter survey only. A plausible reason for this could be nomadic movement of lorikeets to track nectar at large scales and can be influenced by phenology, or regular seasonal shifts [21, 43]. Our results align with others who have suggested increasing population density of aggressive nectar-eating birds (such as Noisy Miner) can lead to interspecies competition, and species displacement, particularly of small nectarivores and insectivores, leading to reductions in urban biodiversity [47, 48, 49]. Indeed, Noisy Miners are considered to be a dominant force structuring urban bird communities [50].

This study revealed that two species (Red Wattlebird and Australian Magpie) contributed to differences between urban and rural assemblages in different directions depending on bioregion and season. There may be several reasons for these patterns including differences in species assemblages meaning that the same species occupy different niches in different bioregions or climatic differences between bioregions may alter the reliance on bird baths as a water source. The life history traits of these birds may enable them to exploit resources in both urbanisation types, and garden habitat may positively influence the presence of these birds [51, 52, 53, 54]. Red Wattlebirds, Australian Magpies and Noisy Miners could be termed ‘urban adaptors’ [3, 11] as they can adapt and exploit resources in the urban landscape and maintain populations in rural areas. While we have speculated on possible reasons for these patterns more research is needed to fully explore these findings.

Birds that are sensitive to urban disturbance and intolerant or unable to use the urban matrix are termed urban avoiders [3, 11]. A number of small insectivorous birds (Superb Fairy-wrens, Grey Fantails), seed-eating birds (Double-barred and Red-browed Finch) and honeyeaters (Lewin’s and New Holland Honeyeaters, Eastern Spinebills) were recorded more frequently at rural bird baths in eight bioregions. Superb Fairy-wrens were recorded in high abundance at rural bird baths in five bioregions. While small insectivorous, seed-eating birds and small honeyeaters were more frequently recorded in rural areas they did appear in low occurrence at urban bird baths in a number of bioregions. For example, Eastern Spinebills were recorded in similar proportions at urban and rural bird baths in South Eastern Highlands. Thus these birds do not appear to be true ‘urban avoiders’ as defined by [11]. The gregarious, aggressive and/or cooperative breeding behaviour of these small birds may help in giving them a competitive advantage when they occur at urban bird baths and help them persist in gardens and use baths. In addition the gardens where these birds were recorded visiting baths may have garden characteristics (native vegetation, dense shrub layer) that help these birds persist. Other factors that may affect birds visiting baths (e.g. streetscape vegetation, nomadic/seasonal migration, supplementary feeding, proportion of native plants and presence of cats and dogs in gardens) warrant further investigation.

Our results demonstrate that different assemblages of birds visit bird baths in gardens/yards depending on bioregion and degree of urbanisation. We report higher species richness at rural bird baths, where a mix of small nectar-eating birds, insectivores and seed-eating birds occurred compared to the more homogenised bird assemblages at urban bird baths.

The data used in this study could have only been collected with the support and enthusiasm of citizen scientists as we required a substantial amount of data from private gardens across an array of bioregions. Although we experienced a number of issues with the contributors (e.g. incomplete survey effort resulting in much data discarded from analysis, low participation rate in a number of bioregions), the scale of this study was only possible through their efforts. Thus, this study illustrates some of the benefits of working with citizen scientists in collecting wildlife survey data and its use for understanding human behaviour (such as the provision of water) [55].

## Supporting Information

S1 File Supporting Information. (XLSX)
